# Cardiovascular involvement in ankylosing spondylitis and axial spondyloarthritis: epidemiology, mechanisms, and clinical management

**DOI:** 10.3389/fmed.2026.1841913

**Published:** 2026-05-18

**Authors:** Xiaoxi Dai, Qi Zhao

**Affiliations:** Department of Rheumatology and Immunology, Shengjing Hospital of China Medical University, Shenyang, China

**Keywords:** ankylosing spondylitis, axial spondyloarthritis, cardiovascular disease, endothelial dysfunction, inflammation

## Abstract

Ankylosing spondylitis (AS) and axial spondyloarthritis (axSpA) are chronic inflammatory diseases primarily affecting the axial skeleton. Increasing evidence indicates that their impact extends beyond the musculoskeletal system and includes clinically relevant cardiovascular involvement. Cardiovascular disease has become an important determinant of long-term outcomes in these patients, with growing evidence showing increased risks of myocardial infarction, stroke, atrial fibrillation, and other major adverse cardiovascular events. In addition to overt clinical events, subclinical vascular abnormalities, endothelial dysfunction, arterial stiffness, and structural cardiac changes are frequently observed. Cardiovascular injury in this setting arises from a complex interplay between traditional risk factors and persistent systemic inflammation. Inflammatory pathways involving tumor necrosis factor, interleukin-17, and interleukin-6 appear to promote endothelial activation, oxidative stress, lipid dysfunction, vascular remodeling, and atherosclerosis. Current evidence suggests that tumor necrosis factor inhibitors may provide cardiovascular benefit through improved control of inflammation, whereas the long-term cardiovascular effects of interleukin-17 inhibitors, non-steroidal anti-inflammatory drugs, and Janus kinase inhibitors remain incompletely defined. These findings support a more integrated clinical approach in AS/axSpA, combining disease control with cardiovascular risk assessment, modification of conventional risk factors, and ongoing cardiovascular surveillance.

## 1 Introduction

Ankylosing spondylitis (AS) and axial spondyloarthritis (axSpA) comprise a disease spectrum characterized primarily by chronic inflammation of the spine and sacroiliac joints. They usually begin in young adulthood, follow a prolonged disease course, and may lead to structural damage progression, impaired physical function, and substantial comorbidity burden over time (1–3). For many years, both clinical practice and research focused mainly on pain, stiffness, restricted mobility, radiographic progression, and disability. However, accumulating evidence from modern imaging, biomarker, and longitudinal outcome studies has increasingly highlighted the systemic nature of AS/axSpA (4–6). As immune-mediated inflammatory diseases, their pathologic processes involve not only entheses, synovium, and bone, but also the vascular wall, myocardium, heart valves, and metabolic organs through persistent systemic inflammation (4–7).

In other inflammatory rheumatic diseases, particularly rheumatoid arthritis, the concept that chronic inflammation accelerates atherosclerosis has long been widely accepted (5, 7). In AS/axSpA, by contrast, cardiovascular risk has historically been underestimated, partly because patients are often younger, more frequently male, and less likely to receive long-term glucocorticoids, thus seeming not to fit the classic high-risk metabolic profile (5, 6, 8). Nevertheless, increasing evidence indicates that being young does not necessarily mean being at low cardiovascular risk. Patients with AS/axSpA have been reported to exhibit endothelial dysfunction, arterial stiffness, and early atherosclerotic changes even before overt cardiovascular symptoms become clinically apparent (9–13). Over time, these subclinical abnormalities may contribute to an increased risk of major cardiovascular outcomes, including stroke, atrial fibrillation, heart failure, and valvular heart disease (6, 14–17).

Furthermore, the cardiovascular burden of AS/axSpA is markedly heterogeneous (2, 3, 18, 19). Patients with persistently high inflammatory activity, long disease duration, suboptimal disease control, or extra-articular manifestations such as uveitis, psoriasis, or inflammatory bowel disease may carry a particularly increased cardiovascular risk (18–21). Pharmacologic therapy does not merely relieve musculoskeletal symptoms; it may also influence future vascular and cardiac outcomes (22–26). Reassessing cardiovascular risk in AS/axSpA is therefore not simply an epidemiologic exercise, but a necessary step toward updating the overall management paradigm for these diseases (4–6, 26).

**Figure 1:**
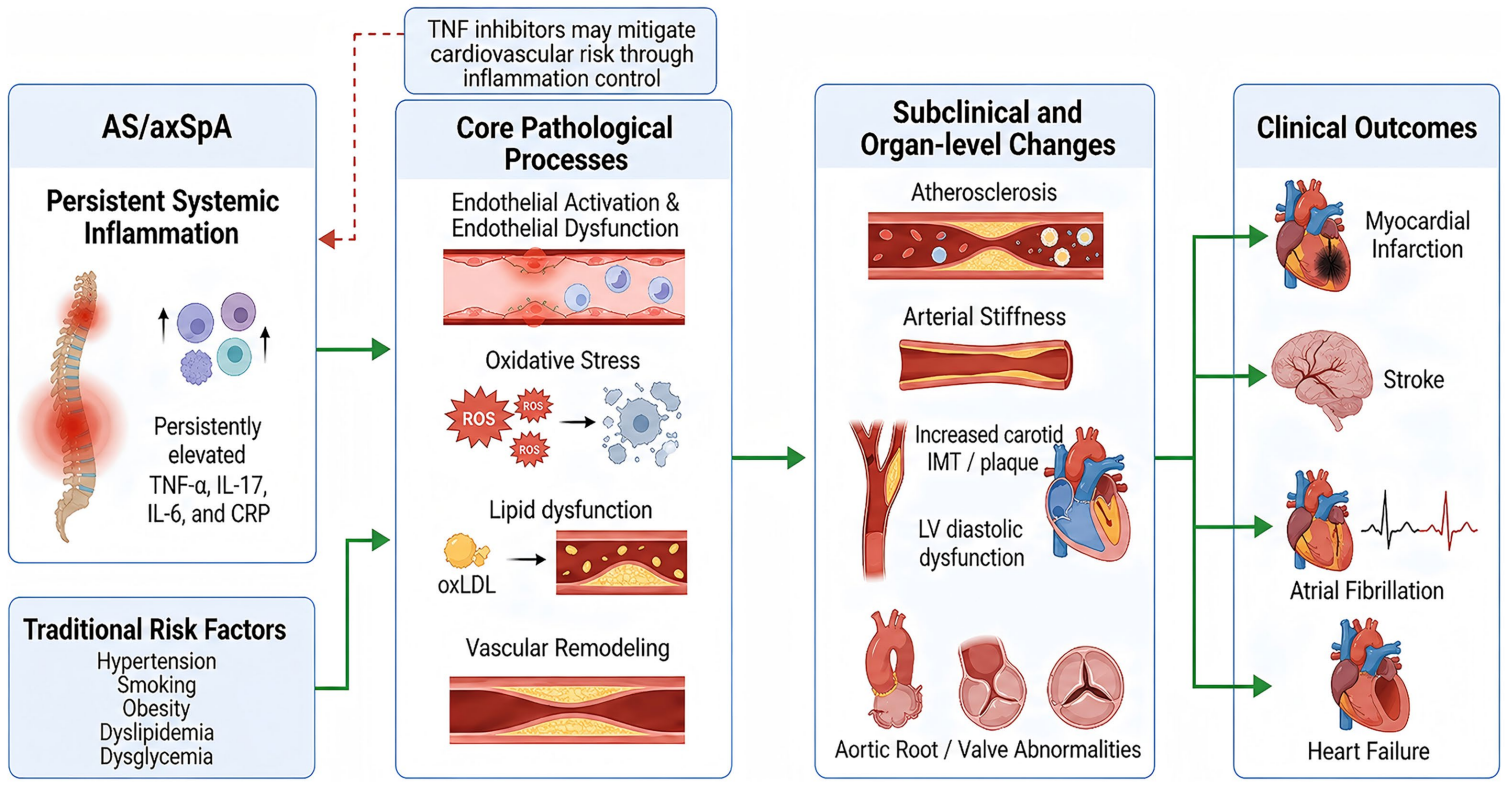
Overview of the core mechanisms underlying cardiovascular involvement in ankylosing spondylitis and axial spondyloarthritis. Persistent systemic inflammation and traditional cardiovascular risk factors jointly promote endothelial activation, oxidative stress, lipid dysfunction, and vascular remodeling, leading to subclinical vascular and cardiac changes such as atherosclerosis, arterial stiffness, increased carotid intima-media thickness or plaque, left ventricular diastolic dysfunction, and aortic root or valvular abnormalities. These changes may ultimately contribute to clinical cardiovascular outcomes, including myocardial infarction, stroke, atrial fibrillation, and heart failure. The dashed red line indicates that TNF inhibitors may mitigate cardiovascular risk indirectly through improved inflammation control. AS, ankylosing spondylitis; axSpA, axial spondyloarthritis; CRP, C-reactive protein; IL, interleukin; IMT, intima-media thickness; LV, left ventricular; TNF, tumor necrosis factor; oxLDL, oxidized low-density lipoprotein; ROS, reactive oxygen species.

This review summarizes the epidemiologic evidence for cardiovascular risk in AS/axSpA, discusses subclinical atherosclerosis and vascular dysfunction, reviews cardiac structural abnormalities and valvular disease, examines the distinctive features of traditional risk factors and metabolic comorbidities in this population, outlines the inflammatory and immune mechanisms that drive cardiovascular injury, evaluates the potential effects of antirheumatic therapies on cardiovascular outcomes, and highlights integrated management strategies and priorities for future research. The overall framework linking chronic inflammation, vascular injury, subclinical cardiovascular abnormalities, and overt cardiovascular outcomes in AS/axSpA is summarized in Figure 1.

## 2 Methods

This narrative review was designed to synthesize current evidence on cardiovascular involvement in AS/axSpA, including epidemiology, subclinical vascular injury, cardiac structural abnormalities, inflammatory and immune mechanisms, pharmacologic influences, and clinical management. Literature searches were performed in PubMed, Embase, and Web of Science from database inception to January 31, 2026. Searches were restricted to English-language publications in humans. The search strategy combined disease-related terms, including “ankylosing spondylitis,” “axial spondyloarthritis,” “radiographic axial spondyloarthritis,” “non-radiographic axial spondyloarthritis,” and “axSpA,” with cardiovascular terms. Mechanistic and treatment-related terms were added in supplementary topic-specific searches where appropriate, rather than used as mandatory filters for all searches. Representative database-specific search strings are provided in Supplementary Table S1.

Eligible publications included systematic reviews, meta-analyses, large population-based cohort studies, registry studies, multicenter observational studies, longitudinal imaging studies, and representative mechanistic or biomarker studies directly relevant to cardiovascular manifestations in AS/axSpA. Studies were considered relevant if they addressed clinical cardiovascular outcomes, all-cause or cardiovascular mortality, subclinical vascular abnormalities, cardiac structural or functional abnormalities, cardiovascular risk factors, inflammatory mechanisms, or cardiovascular effects and safety of antirheumatic therapies. Studies focusing on non-AS/axSpA populations were included only when AS/axSpA-specific evidence was unavailable and when the data provided necessary context for cardiovascular mechanisms, treatment safety, or risk-management principles; such extrapolations were interpreted cautiously.

Publications were excluded if they were not available in English, were not conducted in humans, had no direct relevance to cardiovascular involvement or cardiovascular risk in AS/axSpA, or consisted of isolated case reports that did not contribute to the main epidemiologic, mechanistic, or clinical arguments of the review. Editorials, narrative commentaries, conference abstracts without sufficient methodological detail, and duplicate reports were not used as primary evidence. When several publications addressed overlapping populations or outcomes, preference was given to the most recent, largest, or methodologically robust study.

Study selection was performed in two stages. Titles and abstracts were first screened for relevance, followed by full-text assessment of potentially eligible articles. The reference lists of key reviews, meta-analyses, and major cohort studies were also manually screened to identify additional relevant publications. Evidence was selected and interpreted according to topic relevance, study design, sample size, adjustment for conventional cardiovascular risk factors, outcome ascertainment, follow-up duration, and consistency with the wider literature.

Because this article was a narrative review rather than a systematic review, no prespecified protocol, formal PRISMA flow diagram, duplicate quantitative data extraction, formal risk-of-bias scoring, or pooled meta-analysis was performed. The evidence was synthesized descriptively to provide an integrated and clinically oriented overview of cardiovascular involvement in AS/axSpA. Where available, quantitative estimates from meta-analyses, population-based studies, and longitudinal cohorts were prioritized and are summarized in the relevant tables.

## 3 Epidemiologic risk of cardiovascular disease in AS/axSpA

### 3.1 Overall cardiovascular event risk is increased

The clearest evidence comes from population-based database studies and meta-analyses. Pooled data indicate that patients with spondyloarthritis, including AS/axSpA, have a significantly increased risk of major cardiovascular events compared with the general population, with excess risks particularly evident for myocardial infarction and stroke (5, 6, 16, 27, 28). These findings suggest that, even in the era of modern treatment, AS/axSpA-related inflammation continues to impose a sustained threat to the vasculature. More recent studies further suggest that, although biologic therapies may mitigate cardiovascular risk, inflammatory arthritis remains associated with higher cardiovascular event rates than those seen in the general population, implying that excess inflammation-driven risk has not been fully eliminated by therapeutic advances (22–26).

This conclusion is clinically important. It indicates that cardiovascular risk in AS/axSpA is not merely a historical residue from periods of inadequate treatment, but a genuine complication burden that still exists in current practice (22, 24–26). In other words, even if pain and stiffness are partially relieved, clinicians should not assume that long-term cardiovascular risk has returned to the level of the general population.

### 3.2 Beyond ischemic events: atrial fibrillation, heart failure, and valvular disease

In terms of the event spectrum, coronary artery disease and cerebrovascular disease are among the most prominent clinical manifestations. Available data suggest that the risk of myocardial infarction in patients with AS/axSpA is approximately 1.3–1.6 times that of the general population, whereas the risk of stroke is also significantly increased (6, 16, 28–31). These findings indicate that cardiovascular injury in AS/axSpA is not limited to isolated case reports, but constitutes a quantifiable and reproducible excess burden of ischemic events.

Beyond classic atherosclerotic outcomes, atrial fibrillation is also increasingly recognized in AS/axSpA and other inflammatory arthritides. Systematic reviews and contemporary overviews have reported a higher risk of atrial fibrillation in inflammatory arthritis compared with controls, even after accounting for traditional cardiovascular risk factors (15, 32, 33). This suggests that AS/axSpA-related inflammation may increase cardiovascular risk not only by promoting coronary atherosclerosis, but also by facilitating atrial structural and electrical remodeling, inflammation-mediated fibrosis, and autonomic imbalance. Meanwhile, several population-based studies suggest an increased risk of heart failure in immune-mediated inflammatory disease, and myocardial dysfunction in AS/axSpA appears to be clinically relevant, particularly when disease duration is long or vascular load is increased (14, 17, 34).

Accordingly, cardiovascular risk in AS/axSpA should not be narrowly understood as susceptibility to coronary artery disease alone. Rather, it should be viewed as a broader spectrum of organ outcomes encompassing ischemic events, arrhythmias, valvular disease, and heart failure (6, 15, 17, 34, 35). Screening and follow-up strategies should be broadened accordingly.

### 3.3 All-cause mortality remains debated, but cardiovascular mortality is clearly important

Evidence for increased all-cause mortality in AS/axSpA is less consistent than evidence for increased cardiovascular morbidity. Some meta-analytic data suggest a possible increase in mortality, whereas other studies report weaker or non-significant associations (6, 8). This inconsistency likely reflects the relatively young age of many AS/axSpA cohorts, variable follow-up duration, treatment-era effects, competing causes of death, and differences in adjustment for comorbidities and conventional cardiovascular risk factors.

Nevertheless, the lack of a uniform all-cause mortality signal should not be interpreted as evidence that cardiovascular prevention is unnecessary. Cardiovascular disease remains an important contributor to adverse long-term outcomes in AS/axSpA, and cause-specific analyses continue to support attention to cardiovascular morbidity and cardiovascular mortality (4, 6, 8). Therefore, cardiovascular prevention in AS/axSpA should be justified primarily by the reproducible excess burden of cardiovascular events and organ damage, rather than by all-cause mortality data alone.

### 3.4 Risk is heterogeneous: persistent inflammation, extra-articular manifestations, and difficult-to-treat disease identify high-risk phenotypes

The cardiovascular risk of individual patients with AS/axSpA is clearly heterogeneous. Patients with sustained high disease activity, prolonged inflammatory burden, multiple comorbidities, longer disease duration, or extra-articular manifestations often appear to be at higher risk (2, 3, 18, 19, 21). One particularly notable clue is ocular inflammatory involvement. Population-based studies have shown that patients with AS/axSpA and uveitis have a significantly higher risk of acute coronary syndrome and stroke than those without uveitis, suggesting that ocular involvement may identify a subgroup with more pronounced systemic inflammatory burden (20, 36–38). More broadly, evidence from other immune-mediated diseases with ocular involvement also supports the concept that extra-articular inflammatory phenotypes may cluster with greater systemic morbidity and mortality, although such data should be interpreted as supportive rather than disease-specific for AS/axSpA (39).

This observation indicates that uveitis is not merely an ophthalmic complication, but may reflect a state of greater systemic inflammatory activation. Similarly, axial spondyloarthritis accompanied by psoriasis, inflammatory bowel disease, or multiple comorbidities may carry an additional cardiovascular burden (2, 18, 19). Difficult-to-treat axial spondyloarthritis, characterized by high disease activity, comorbidity clustering, and poor therapeutic response, may therefore represent an especially important subgroup for intensified cardiovascular monitoring (2, 3). Representative epidemiologic studies supporting the excess cardiovascular risk in AS/axSpA are summarized in Table 1.

**Table 1:** Subclinical vascular abnormalities in ankylosing spondylitis and axial spondyloarthritis.

Abnormality	Assessment	Representative quantitative evidence	Evidence strength	Clinical relevance
Increased carotid intima-media thickness	Carotid ultrasonography	Law et al. (10): 155 r-axSpA patients versus 400 controls; mean cIMT 0.8 +/− 0.1 mm versus 0.7 +/− 0.1 mm, adjusted *p* < 0.001	Moderate	Supports accelerated or premature subclinical atherosclerotic change
Carotid plaque	Carotid ultrasonography	Gonzalez Mazon et al. (41): 806 axSpA patients (639 AS, 167 nr-axSpA); after adjustment, plaque prevalence and cIMT did not differ substantially between AS and nr-axSpA	Moderate	Vascular injury is not confined to long-standing radiographic disease.
Endothelial dysfunction	Flow-mediated dilation and microvascular endothelial testing	Cuesta-Lopez et al. (9): 40% of axSpA patients had sustained CRP elevation and showed worse endothelial function, more metabolic comorbidity, and greater plaque burden	Moderate	May represent an early functional stage before overt events
Increased arterial stiffness	Pulse wave velocity and stiffness indices.	Arida et al. (11) and related longitudinal/imaging studies indicate measurable progression over years; inflammatory burden and conventional risk factors both contribute.	Moderate	Links vascular inflammation with premature vascular aging and increased ventricular afterload
Increased epicardial adipose tissue thickness	Echocardiography.	Demir et al. (13): AS patients showed higher epicardial adipose tissue thickness and arterial stiffness parameters than controls in a subclinical atherosclerosis assessment.	Low to moderate	Reflects inflammatory-metabolic interaction around the heart and coronary circulation
Soluble endothelial biomarker abnormalities	Serum biomarker assays	Pusztai et al. (46): 1-year anti-TNF-alpha therapy altered soluble vascular biomarkers in AS and RA, supporting treatment-responsive endothelial activation pathways	Low to moderate	Potential adjunctive marker set for vascular injury, but not ready for routine risk prediction.
Syndecan-related abnormalities	Serum syndecan assays	Sertdemir et al. (47) and Yilmaz et al. (49) linked syndecan-family markers with subclinical atherosclerosis measures in AS	Low	Hypothesis-generating biomarkers for vascular risk stratification
Progressive subclinical atherosclerosis	Longitudinal carotid ultrasound and vascular assessment	Arida et al. (11): 10-year prospective data support progression of subclinical atherosclerosis in AS over time	Moderate	Supports early prevention rather than waiting for clinical cardiovascular events

AS, ankylosing spondylitis; axSpA, axial spondyloarthritis; cIMT, carotid intima-media thickness; CRP, C-reactive protein; nr-axSpA, non-radiographic axial spondyloarthritis; r-axSpA, radiographic axial spondyloarthritis; TNF, tumor necrosis factor. Evidence strength was graded pragmatically for this narrative review: high = meta-analysis or large population-based cohort with adjusted estimates; moderate = multicenter cohort, registry study, or consistent observational evidence; low = single-center, surrogate-only, short-term, or indirect evidence.

### 3.5 Sex-specific considerations in cardiovascular risk

Sex-specific cardiovascular risk in AS/axSpA remains insufficiently characterized. Although AS/axSpA, particularly radiographic disease, is more frequently diagnosed in men, cardiovascular risk should not be considered a male-specific issue. Women with axSpA may experience different symptom patterns, longer diagnostic delay, different disease burden, and distinct comorbidity profiles, all of which may influence cardiovascular risk recognition, treatment exposure, and preventive care (4, 40). Available epidemiologic studies are not sufficiently harmonized to support sex-specific cardiovascular risk thresholds in AS/axSpA. Nevertheless, future studies should report cardiovascular events and vascular surrogate markers stratified by sex and should adjust for sex-related differences in smoking, obesity, metabolic comorbidities, disease phenotype, extra-articular manifestations, and treatment exposure.

## 4 Subclinical atherosclerosis and vascular dysfunction

### 4.1 Subclinical abnormalities often precede clinical events

Cardiovascular injury in AS/axSpA does not begin only when myocardial infarction or stroke occurs. Rather, subclinical vascular abnormalities are frequently present before overt cardiovascular events develop (7, 10–13). Consequently, reliance on a prior history of coronary artery disease or stroke alone will often underestimate the true severity of the problem.

The major importance of the subclinical stage is that it reveals a continuum linking inflammation, vascular injury, and clinical events. Many relatively young patients with AS/axSpA have no typical chest pain or transient ischemic attack symptoms, and routine laboratory testing may not show striking metabolic abnormalities, yet structural and functional vascular alterations are already detectable on imaging and physiologic assessment (10–13). Recognizing this stage helps shift the preventive window earlier.

### 4.2 Increased carotid intima-media thickness and plaque formation

Carotid ultrasonography is a classic method for assessing subclinical atherosclerosis. Studies have shown that patients with radiographic axial spondyloarthritis have significantly greater carotid intima-media thickness than matched controls, supporting an increased atherosclerotic burden even before overt events occur (10, 12, 41).

Beyond IMT thickening, plaque formation is also common. In a large multicenter study, carotid plaque was detected in a substantial proportion of patients, and no major difference in plaque prevalence or IMT was observed between radiographic AS and non-radiographic axSpA (19, 41). This is important because it suggests that even in earlier disease stages without typical radiographic structural damage, patients may already harbor an atherosclerotic burden comparable to that of established radiographic AS. Vascular injury therefore appears to be driven more by inflammatory activity and cumulative risk exposure than by skeletal damage alone (11, 19, 41).

### 4.3 Increased arterial stiffness: elevated pulse wave velocity reflects premature vascular aging

Pulse wave velocity is an important marker of large-artery stiffness. Studies comparing patients with AS/axSpA and controls, including those minimizing the influence of major traditional risk factors, have found significantly greater arterial stiffness in AS/axSpA (7, 13, 42, 43). This implies that even in the absence of overt metabolic cardiovascular disease, patients with AS/axSpA may exhibit substantially reduced arterial elasticity, resembling a state of premature vascular aging.

The significance of arterial stiffness extends beyond the arteries themselves. Stiffer large arteries increase systolic load and pulse pressure, thereby promoting left ventricular remodeling, diastolic dysfunction, and eventually heart failure (7, 44). In this sense, arterial stiffness forms an important bridge between vascular and cardiac pathology. Within inflammatory arthritis more broadly, arterial stiffness also correlates with conventional cardiovascular risk, indicating that inflammation and traditional risk factors act together rather than as two independent systems (7, 43, 44).

### 4.4 Increased epicardial adipose tissue thickness highlights the interplay between inflammation, metabolism, and the vasculature

Patients with AS/axSpA have also been reported to exhibit greater epicardial adipose tissue thickness than controls (13). Epicardial adipose tissue is increasingly viewed as a marker of visceral adiposity and systemic inflammation and has been linked to coronary inflammation, metabolic disturbance, and atherosclerosis (4, 13, 45). In AS/axSpA, increased epicardial adipose tissue suggests that the disease involves not only inflammation of the vascular wall itself, but also broader alterations in the metabolic-inflammatory microenvironment surrounding the heart.

### 4.5 Endothelial dysfunction: an early hallmark of atherosclerosis

Compared with IMT and PWV, endothelial dysfunction may represent an even earlier and more functional abnormality. Flow-mediated dilation is impaired in AS/axSpA, and several vascular biomarker studies have also demonstrated endothelial activation and injury (9, 12, 46). At the same time, soluble adhesion molecules and other endothelial injury markers are elevated, indicating endothelial activation, damage, and a proatherogenic vascular phenotype (9, 46–48). Additional work suggests that syndecan-related pathways may also be relevant, because circulating syndecan-family markers have been linked to subclinical vascular injury and may help refine early risk stratification in AS/axSpA (47, 49).

Endothelial dysfunction implies reduced nitric oxide bioavailability, impaired vasodilatory capacity, enhanced leukocyte adhesion to the vascular wall, and increased susceptibility to intimal lipid deposition, thereby creating the microenvironment for subsequent plaque formation. Importantly, these abnormalities are associated with disease activity, duration, and sustained inflammatory burden, supporting the view that endothelial injury is closely linked to chronic inflammation rather than merely coexisting by chance (9, 12, 48, 49).

### 4.6 Long-term progression: traditional risk factors provide the substrate, while inflammation acts as an accelerator

Longitudinal studies suggest that both patients with AS/axSpA and controls may show progression of atherosclerosis over time, and that traditional factors such as age, smoking, blood pressure, and lipid-related risk remain important determinants of progression (11, 19, 50, 51). In multivariable models, a diagnosis of AS/axSpA itself is not always the only independent driver. However, within the AS/axSpA population, patients with poorer disease control and higher inflammatory burden show more pronounced progression of vascular abnormalities (11, 19, 21).

This is a particularly important point for discussion. Cardiovascular injury in AS/axSpA is not a completely separate process detached from the general biology of atherosclerosis. Traditional risk factors provide the biological substrate, whereas persistent inflammation acts as an accelerator that hastens the transition from slowly evolving arterial disease to clinically meaningful pathology (11, 19, 21, 50). Consequently, controlling inflammation alone or controlling blood pressure and lipids alone is insufficient; both are necessary. The main forms of subclinical vascular injury reported in AS/axSpA are summarized in Table 1.

## 5 Cardiac structural abnormalities and valvular disease

### 5.1 Aortic root and aortic valve disease are classic cardiac manifestations of AS/axSpA

Among the cardiac manifestations associated with AS/axSpA, aortic root involvement and secondary valvular abnormalities are among the most characteristic. Earlier pathophysiologic observations and more recent systematic analyses support an association between axial spondyloarthritis and aortic or left-sided valvular abnormalities, particularly aortic regurgitation (17, 35, 52). These findings support the concept that AS/axSpA is associated not merely with occasional valvular involvement, but with a meaningful increase in the risk of structural cardiac disease. In parallel, broader studies of aortitis and periaortitis outside AS/axSpA provide a useful contextual framework, indicating that inflammatory disease of the aortic wall can follow a clinically relevant but heterogeneous course and may contribute to later valvular or aortic root abnormalities (53, 54).

### 5.2 The natural history of aortic regurgitation is often slow but clinically relevant

Follow-up echocardiographic data suggest that AS/axSpA-related aortic regurgitation is often mild and may progress slowly over the medium term (17, 52). This is clinically important. On the one hand, the detection of mild or moderate aortic regurgitation should not automatically lead to the assumption of rapid deterioration; on the other hand, apparently slow short-term progression should not justify abandoning follow-up. Continued echocardiographic surveillance remains reasonable, particularly in patients with long disease duration or pre-existing aortic root abnormality (52).

### 5.3 HLA-B27 may partly contribute to the aortic root phenotype

Some studies have suggested that HLA-B27 positivity may be linked to aortic root abnormalities in selected patients, although the available evidence remains limited and is not yet sufficient to support inclusion of HLA-B27 as an independent cardiovascular risk predictor (17, 52). At present, HLA-B27 may be viewed as a potentially useful stratification signal in selected patients undergoing evaluation for aortic root involvement rather than as a definitive cardiovascular risk marker. More generally, the broader clinical spectrum of inflammatory aortitis and periaortitis supports the idea that chronic immune activation may shape distinct aortic phenotypes, even though AS/axSpA-specific data remain more limited (53, 54).

### 5.4 Left ventricular diastolic dysfunction appears more common than systolic dysfunction

Beyond valvular abnormalities, myocardial involvement in AS/axSpA more commonly manifests as diastolic dysfunction than as overt systolic impairment. A recent meta-analysis of echocardiographic studies found only minor differences in left ventricular ejection fraction between patients and controls, whereas indices of diastolic function showed more consistent abnormalities (17, 34). Assessment of diastolic dysfunction should therefore not rely on ejection fraction alone. Relevant echocardiographic parameters include mitral inflow E/A ratio, tissue Doppler e’ velocity, E/e’ ratio, left atrial volume index, tricuspid regurgitation velocity, isovolumic relaxation time, and grading according to contemporary echocardiographic recommendations. Preserved left ventricular ejection fraction does not exclude early myocardial involvement.

Mechanistically, chronic inflammation-induced interstitial fibrosis, pressure overload related to arterial stiffness, hypertension, and microvascular dysfunction may all contribute to impaired myocardial relaxation (7, 34, 44). Over time, these abnormalities may translate into reduced exercise tolerance, dyspnea, heart failure with preserved ejection fraction, or other clinically apparent heart failure phenotypes.

### 5.5 Clinical echocardiographic studies and epidemiologic studies do not always align

Small echocardiographic studies and large epidemiologic analyses do not always yield fully consistent conclusions regarding the prevalence of valvular abnormalities. Limited sample sizes, younger study populations, and the low frequency of clinically important valvular disease may reduce the power of single-center echocardiographic studies, whereas larger epidemiologic datasets are better positioned to capture cumulative long-term risk (17, 35, 52). This discrepancy should not be interpreted as evidence against population-level findings. A more plausible interpretation is that cardiac involvement in AS/axSpA accumulates over time and may begin as subclinical structural change before becoming clinically apparent valvular disease in older patients with longer-standing disease (17, 52). Cardiac structural abnormalities and clinical manifestations associated with AS/axSpA are summarized in Table 2.

**Table 2:** Cardiac structural abnormalities and clinical manifestations in ankylosing spondylitis and axial spondyloarthritis.

Manifestation	Representative quantitative evidence	Diagnostic parameters	Evidence strength	Clinical relevance
Aortic root involvement	Classic AS-associated manifestation, but quantitative estimates vary across cohorts. Bengtsson et al. (52) followed a selected subgroup with and without AR over 3–5 years.	Echocardiographic aortic root and proximal ascending aorta diameter; assessment of cusp thickening/retraction when present.	Low to moderate	May precede or contribute to aortic regurgitation; surveillance should be individualized.
Aortic regurgitation/valvular heart disease	Romand et al. (17): 28 studies, 1,471 axSpA patients and 1,115 controls; mitral and aortic regurgitation prevalence was similar to controls. Bengtsson et al. (52): 26 baseline AR cases included 18 mild, 7 moderate, and 1 severe case; most remained unchanged at follow-up.	Transthoracic echocardiography with severity grading and aortic root measurement.	Moderate	Usually slow in available follow-up data, but clinically relevant in long-standing disease or abnormal examination.
Left ventricular diastolic dysfunction	Romand et al. (17): LV diastolic dysfunction was more frequent in axSpA (OR 3.43, 95% CI 1.78–6.59); altered E/A ratio (MD 0.15), deceleration time (MD 13.07 ms), and IVRT (MD 7.90 ms).	E/A ratio, tissue Doppler e-prime, E/e-prime, left atrial volume index, tricuspid regurgitation velocity, and integrated diastolic grading.	Moderate to high	Preserved LVEF does not exclude early myocardial involvement.
Left ventricular systolic dysfunction	Romand et al. (17): LVEF difference was statistically small (MD 0.64, 95% CI 0.14–1.14) and considered not clinically relevant. Bolaji et al. (34) further supports that early dysfunction is often not captured by LVEF alone.	LVEF, LV dimensions, and preferably myocardial strain where available.	Moderate	Less consistently abnormal than diastolic indices; strain or diastolic parameters may detect earlier involvement.
Atrial fibrillation and rhythm disorders	Ma et al. (33): inflammatory arthritis was associated with increased AF risk (pooled HR 1.42, 95% CI 1.36–1.49; adjusted HR 1.37, 95% CI 1.29–1.46). Konstantinou et al. (15) provides an AS-focused overview supporting increased AF susceptibility.	ECG for symptoms or rhythm concern; ambulatory rhythm monitoring if palpitations, syncope, or unexplained dyspnea occur.	Moderate	Expands the cardiovascular phenotype beyond coronary and cerebrovascular disease, while avoiding unsupported extrapolation to conduction-specific outcomes.
Heart failure risk	Koppikar et al. (14): IMID cohort had crude HF incidence 2.70 per 1,000 person-years; r-axSpA had the lowest HF rate among IMID groups. Echocardiographic evidence (17, 34) supports clinically relevant myocardial functional abnormalities, especially diastolic dysfunction, that may contribute to HF phenotypes.	Clinical assessment, natriuretic peptides when indicated, echocardiography, and cardiology work-up for dyspnea or reduced exercise tolerance.	Moderate	Risk is likely amplified by arterial stiffness, hypertension, diastolic dysfunction, and multimorbidity.

AF, atrial fibrillation; AR, aortic regurgitation; AS, ankylosing spondylitis; axSpA, axial spondyloarthritis; CI, confidence interval; ECG, electrocardiography; HF, heart failure; HR, hazard ratio; IMID, immune-mediated inflammatory disease; IVRT, isovolumetric relaxation time; LV, left ventricular; LVEF, left ventricular ejection fraction; MD, mean difference; OR, odds ratio; r-axSpA, radiographic axial spondyloarthritis. Evidence strength was graded pragmatically for this narrative review: high = meta-analysis or large population-based cohort with adjusted estimates; moderate = multicenter cohort, registry study, or consistent observational evidence; low = single-center, surrogate-only, short-term, or indirect evidence.

## 6 Traditional cardiovascular risk factors and metabolic comorbidities

### 6.1 Hypertension may not appear strikingly prevalent, but it should not be overlooked

Compared with rheumatoid arthritis or psoriatic arthritis, hypertension in AS/axSpA may appear less prominent at first glance, partly because AS/axSpA populations are younger on average. However, this does not mean that blood pressure-related risk is minor. Real-world data suggest that hypertension and blood pressure-related organ damage are clinically relevant in AS/axSpA, especially with increasing age and cumulative disease burden (4, 21, 50).

More importantly, subclinical blood pressure abnormalities matter. Even in patients without overt cardiovascular disease, ambulatory blood pressure monitoring has demonstrated abnormal nocturnal dipping patterns, suggesting dysregulated vascular control and early cardiovascular stress (51). Thus, blood pressure-related damage may already be present before formal hypertension is diagnosed. Blood pressure assessment at routine AS/axSpA visits should therefore be standard practice rather than postponed until patients become older or manifest overt cardiovascular disease (50, 51).

### 6.2 Dyslipidemia: the inflammatory lipid paradox is central to metabolic risk in AS/axSpA

The lipid profile of patients with AS/axSpA cannot be interpreted according to general population logic alone. Under conditions of active inflammation, total cholesterol and LDL-C may not appear markedly elevated, while HDL may decrease and overall lipid function becomes more atherogenic. This is part of the so-called inflammatory lipid paradox (4, 7, 55).

In AS/axSpA, studies have shown that lipid-related pathways are closely linked to inflammatory activity. PCSK9 levels are associated with disease activity, and TNF inhibitor therapy appears to influence lipid-related cardiovascular risk profiles (26, 55). These findings suggest that inflammation disturbs lipid metabolism not merely at the level of concentration, but also at the level of function and pathway regulation. Therefore, clinicians should not be reassured simply because LDL-C is not elevated. Lipid assessment in AS/axSpA should be interpreted alongside inflammatory activity, plaque burden, HDL-related changes, and coexisting cardiovascular risk factors (4, 26, 55).

### 6.3 Abnormal glucose metabolism and metabolic syndrome are common enough to merit attention

A common assumption is that because patients with AS/axSpA rarely receive prolonged glucocorticoids and may appear relatively lean, diabetes risk is low. Yet recent evidence indicates that diabetes is meaningfully more common in AS/axSpA than in the general population (56). Although this excess risk may be smaller than that seen in some other inflammatory arthritides, it remains both statistically and clinically relevant.

Possible mechanisms include chronic inflammation-induced insulin resistance, reduced physical activity, altered body composition, and clustering of metabolic syndrome components. Indeed, metabolic abnormalities are common in AS/axSpA, reinforcing the view that AS/axSpA should not be regarded as metabolically benign (4, 21, 56). This issue becomes even more important in patients with established type 2 diabetes, because immune-mediated inflammatory diseases may further amplify cardiovascular risk in that context (57). Conversely, emerging population-based data suggest that cardiometabolic therapies such as GLP-1 receptor agonists may influence mortality and major adverse cardiovascular events in patients with immune-mediated inflammatory diseases and diabetes, although AS/axSpA-specific interventional evidence is still lacking (58).

### 6.4 Obesity has a dual role: a cardiovascular risk factor and a marker of poorer therapeutic response

The role of obesity in AS/axSpA is complex. On the one hand, not all AS/axSpA populations show a very high prevalence of obesity; on the other hand, excess adiposity promotes inflammatory mediator production, worsens mechanical loading, and may adversely affect disease activity and function (2, 45). Obesity in AS/axSpA is therefore not merely a metabolic issue, but also a contributor to poorer disease control and potentially reduced treatment response.

Accordingly, weight management should be considered part of integrated AS/axSpA care. Improvements in body composition may favorably affect not only blood pressure, glucose and lipid metabolism, but also disease control and physical function (2, 45).

### 6.5 Smoking is a key amplifier of disease and cardiovascular risk

Smoking is among the most important modifiable contributors to cardiovascular risk in AS/axSpA and is also associated with a less favorable disease profile (4, 19, 21). In a review article, this point deserves emphasis because, compared with many inflammatory and genetic determinants that are difficult to modify, smoking is one of the most actionable intervention targets. Smoking cessation may therefore offer dual benefits in AS/axSpA: improved overall disease control and lower long-term cardiovascular risk (4, 19, 21).

### 6.6 Comorbidities increase risk and complicate disease assessment and treatment decisions

Comorbidities not only raise organ risk but also complicate disease assessment. In axSpA cohorts, greater comorbidity burden has been associated with higher disease activity scores and worse overall patient status (2, 3). This is especially important from a cardiovascular perspective because coexisting hypertension, diabetes, depression, and heart failure may simultaneously increase true organ risk, alter symptom burden, affect treatment adherence, and influence medication choice (2–4, 21). Comorbidity patterns may also vary across the wider spondyloarthritis spectrum according to sex and clinical phenotype, suggesting that individualized assessment is preferable to assuming a uniform risk profile for all patients (40).

Comorbidity clustering in chronic inflammatory disease also extends beyond classical cardiometabolic conditions. Although not directly part of cardiovascular risk prediction, studies on hearing disorders and other systemic complications highlight the broader multisystem burden that may accompany chronic inflammatory disease and complicate longitudinal management (59). Cardiovascular comorbidity assessment in AS/axSpA clinics should therefore not be considered ancillary, but central to disease activity interpretation and long-term management. Traditional cardiovascular risk factors and their disease-specific features in AS/axSpA are summarized in Table 3.

**Table 3:** Traditional cardiovascular risk factors and disease-specific features in ankylosing spondylitis and axial spondyloarthritis.

Risk factor	General cardiovascular relevance	Disease-specific feature in AS/axSpA	Clinical implication
Hypertension	Major determinant of cardiovascular morbidity and organ damage	May appear less prominent in younger AS populations, but abnormal blood pressure patterns and organ damage are still relevant	Blood pressure should be assessed routinely rather than deferred because of young age
Dyslipidemia	Important contributor to atherosclerosis	Lipid abnormalities may be modified by active inflammation, including the inflammatory lipid paradox	Lipid interpretation should consider inflammatory burden rather than LDL-C alone
Abnormal glucose metabolism/diabetes	Increases macrovascular and microvascular risk	Diabetes risk appears meaningfully increased in axSpA despite the absence of long-term glucocorticoid exposure in many patients	Glucose metabolism should be screened and managed as part of integrated cardiovascular prevention
Obesity	Promotes hypertension, dyslipidemia, and insulin resistance	Also associated with inflammatory burden, poorer function, and potentially worse treatment response	Weight management may benefit both cardiovascular risk and disease control
Smoking	Strong modifiable cardiovascular risk factor	Also associated with a less favorable AS/axSpA disease profile	Smoking cessation may provide dual benefits for disease activity and cardiovascular prevention
Metabolic syndrome	Increases long-term vascular risk	Metabolic abnormalities may cluster despite the traditional view of AS as metabolically less severe than other rheumatic diseases	Metabolic risk should not be underestimated in AS/axSpA
Comorbidity burden	Amplifies overall cardiovascular risk	Comorbidities may also worsen disease activity scores, symptom burden, and treatment complexity	Cardiovascular assessment should be integrated into routine comorbidity evaluation

AS, ankylosing spondylitis; axSpA, axial spondyloarthritis; LDL-C, low-density lipoprotein cholesterol. In AS/axSpA, conventional cardiovascular risk factors remain important, but their impact is modified by chronic systemic inflammation.

## 7 How inflammatory and immune mechanisms drive cardiovascular injury

### 7.1 Chronic inflammation is the central driver of cardiovascular injury in AS/axSpA

The core of cardiovascular risk in AS/axSpA is not merely the accumulation of traditional risk factors, but the direct vascular and cardiac effects of persistent inflammation. Key mechanistic nodes include TNF-α, IL-6-related pathways, sustained CRP elevation, oxidative stress, endothelial activation, and altered lipoprotein function (6, 7, 9, 25). Thus, cardiovascular pathology in AS/axSpA is not simply the result of hypertension superimposed on aging arteries; rather, it reflects an interconnected inflammatory network that promotes endothelial dysfunction, leukocyte recruitment, vascular remodeling, and myocardial injury (9, 25, 26, 46).

### 7.2 TNF-α links inflammation to atherosclerosis

TNF-α is one of the best supported mediators of vascular injury in AS/axSpA. It upregulates endothelial adhesion molecules, promotes leukocyte recruitment, and impairs nitric oxide-mediated vasodilatory responses, thereby contributing to endothelial dysfunction and vascular remodeling (9, 12, 46). Its pathogenic role is therefore dual: TNF-α not only amplifies inflammation directly, but also weakens endogenous vascular protection.

This helps explain why TNF inhibitor therapy may improve cardiovascular prognosis in parallel with musculoskeletal symptoms. The benefit is unlikely to reflect a direct stand-alone cardioprotective drug effect; rather, it appears to be mediated largely through suppression of systemic inflammation and downstream vascular injury (23–26).

### 7.3 IL-17 may contribute to vascular inflammation, although direct evidence remains less extensive

IL-17 is an important cytokine in AS/axSpA pathogenesis and may also contribute to vascular inflammation. However, compared with TNF-*α*, direct evidence linking IL-17 to cardiovascular injury in AS/axSpA is still less extensive (60–62). At present, IL-17 is best viewed as a plausible contributor to inflammatory cardiovascular risk rather than a fully established mechanistic driver supported by the same level of evidence as TNF-α.

### 7.4 Sustained CRP elevation is not only a disease activity marker but also a signal of organ injury

One of the more notable recent advances concerns the significance of sustained CRP elevation. Persistent or repeatedly elevated CRP levels are associated with endothelial dysfunction, metabolic disturbance, and more adverse vascular phenotypes in axial spondyloarthritis (9). This suggests that CRP is not merely a bystander marker, but a practical indicator of prolonged exposure to an inflammatory, vascular-toxic environment.

From a management perspective, cumulative inflammatory burden may be more informative than a single isolated CRP value. Persistently elevated CRP may therefore help identify patients in whom more intensive cardiovascular prevention and tighter disease control are warranted (9, 10, 25).

### 7.5 IL-6, CDCP1, and PON3 provide emerging molecular links between inflammation and vascular injury

Recent proteomic work has shown that, in patients with sustained inflammatory burden, IL-6 and CDCP1 are increased whereas PON3 is reduced, outlining a more refined molecular link between chronic inflammation and endothelial dysfunction (9). These changes suggest that vascular injury in AS/axSpA is mediated not only by canonical cytokines, but also by altered endothelial adhesiveness, oxidative stress balance, and lipoprotein-associated antioxidant defense.

Notably, these abnormalities appear to improve after effective anti-inflammatory treatment, providing mechanistic support for the vascular benefit of inflammation control (9, 46).

### 7.6 Oxidative stress and impaired lipoprotein function

Chronic inflammation in AS/axSpA is accompanied by oxidative stress and abnormalities in lipoprotein function. Rather than reflecting simply “too much cholesterol,” the lipid milieu becomes more susceptible to oxidation and less capable of vascular protection (9, 46, 55). The vasculature is therefore exposed to the combined insult of oxidative stress, endothelial activation, and chronic inflammation, creating a strongly proatherogenic environment (9, 12, 55).

### 7.7 Immune cells and HLA-B27 may further broaden the mechanistic landscape

Beyond cytokines, broader immune pathways may also contribute to cardiovascular injury in AS/axSpA. Immune cell activation and chronic inflammatory signaling likely influence vascular remodeling and valvular phenotypes, although direct mechanistic evidence remains limited (15, 17, 52). HLA-B27 may also contribute to specific structural cardiac phenotypes, particularly those involving the aortic root, but present evidence is insufficient to conclude that it independently predicts major ischemic events (17, 52).

At a more exploratory level, gut microbiome-related pathways may broaden the mechanistic landscape of systemic organ injury. Microbiome-centered genetic analyses support the broader concept that chronic immune dysregulation can manifest beyond the musculoskeletal axis, although these data should be regarded as hypothesis-generating rather than definitive evidence for cardiovascular mechanisms in AS/axSpA (63). The major inflammatory and immune mechanisms implicated in cardiovascular injury in AS/axSpA are summarized in Table 4.

**Table 4:** Inflammatory and immune mechanisms contributing to cardiovascular injury in ankylosing spondylitis and axial spondyloarthritis.

Mechanism/mediator	Proposed cardiovascular effect	Pathophysiologic consequence	Clinical significance
Chronic systemic inflammation	Sustained vascular and myocardial injury	Endothelial activation, vascular remodeling, myocardial damage	Central driver linking AS/axSpA to excess cardiovascular risk
TNF-alpha	Promotes endothelial activation, leukocyte recruitment, and impaired vasodilation	Endothelial dysfunction and accelerated atherosclerosis	Provides mechanistic rationale for potential cardiovascular benefit of TNF inhibition
IL-17	Contributes to vascular inflammation	May amplify inflammatory vascular injury	Plausible mechanism, but direct evidence remains less extensive than for TNF-alpha
Sustained CRP elevation	Reflects prolonged exposure to inflammatory burden	Associated with endothelial dysfunction and adverse vascular phenotype	May help identify patients requiring tighter disease and cardiovascular risk control
IL-6-related pathways	Promote inflammatory vascular injury	Linked to endothelial dysfunction and chronic vascular stress	Supports the concept that non-TNF cytokine pathways also contribute
CDCP1 and related proteomic signals	May reflect altered endothelial adhesiveness and inflammation	Contribute to endothelial dysfunction	Emerging mechanistic markers requiring further validation
Reduced PON3 and impaired antioxidant defense	Weakens lipoprotein-associated vascular protection	Enhances oxidative stress and proatherogenic change	Highlights the role of dysfunctional lipid biology in inflammatory vascular injury
Oxidative stress	Damages vascular integrity and promotes atherosclerosis	Favors endothelial injury and plaque development	Important link between chronic inflammation and vascular toxicity
Endothelial dysfunction	Reduces nitric oxide bioavailability and impairs vascular homeostasis	Facilitates leukocyte adhesion and intimal lipid deposition	Represents an early, clinically relevant stage of cardiovascular injury
Immune cell activation	Broadens inflammatory vascular and valvular remodeling	May contribute to structural cardiac and vascular phenotypes	Supports a wider immune-mediated model of cardiovascular injury
HLA-B27-related processes	May influence selected structural cardiac phenotypes	Particularly implicated in aortic root abnormalities in some studies	Potential stratification signal, but not an established predictor of major ischemic events
Microbiome-related and non-canonical inflammatory pathways	May contribute indirectly to systemic vascular injury	Broaden the mechanistic landscape beyond classic cytokine pathways	Currently hypothesis-generating rather than definitive

CRP, C-reactive protein; IL, interleukin; PON3, paraoxonase 3; TNF, tumor necrosis factor. Current evidence supports persistent inflammation as the central mechanism of cardiovascular injury in AS/axSpA, while several emerging pathways remain investigational.

## 8 Effects of pharmacologic therapy on cardiovascular outcomes

### 8.1 TNF inhibitors currently have the strongest evidence for cardioprotection

Among currently available AS/axSpA therapies, the evidence most strongly supports a cardiovascular benefit from TNF inhibitors. Meta-analytic and population-based studies suggest that TNF inhibitor exposure is associated with lower cardiovascular event rates in AS/axSpA, particularly when inflammation is effectively suppressed (22–24, 26, 64). This evidence is strengthened by concordant improvements across clinical events, inflammatory burden, and vascular surrogate markers (9, 26, 46).

Importantly, some analyses suggest that the apparent cardioprotective association weakens after adjustment for inflammatory markers, which in fact supports the idea that the benefit is mediated largely through inflammation control (23–26). Clinically, this is highly meaningful: TNF inhibitors are not necessarily direct cardioprotective agents in isolation, but by most effectively reducing chronic systemic inflammation, they may indirectly reduce endothelial dysfunction, plaque progression, and downstream cardiovascular events. At the same time, rare reports of new-onset heart failure in inflammatory joint disease during TNF-α inhibitor treatment and pooled biologic safety analyses remind clinicians that treatment choice should still be individualized, particularly in patients with pre-existing cardiac vulnerability (65, 66). Real-world persistence with TNF inhibitors may also influence the degree to which cardiovascular benefit is sustained over time, although direct cardiovascular endpoint data remain limited (67).

### 8.2 IL-17 inhibitors appear acceptable in the mid-term, but long-term cardiovascular outcomes remain uncertain

IL-17 inhibitors have become important targeted therapies in AS/axSpA. Available pooled analyses and comparative safety studies have not demonstrated a clear major adverse cardiovascular event signal in the short to medium term, but they also have not established definite long-term cardiovascular protection (60, 62, 68). Real-world evidence for secukinumab supports sustained retention, effectiveness, and general safety, although cardiovascular-specific long-term benefit cannot be inferred directly from those data (69). Thus, the most appropriate current conclusion is that IL-17 inhibitors appear to have an acceptable mid-term cardiovascular safety profile, while their long-term effects on hard cardiovascular outcomes remain uncertain.

### 8.3 NSAIDs require individualized cardiovascular risk assessment

NSAIDs are a cornerstone of AS/axSpA treatment and also one of the most controversial drug classes in cardiovascular discussions. In the general population, especially with some agents or dosing patterns, NSAIDs are associated with increased cardiovascular risk. In AS/axSpA, observational studies have reported a more complex association between NSAID exposure and cardiovascular outcomes, with some analyses suggesting neutral or lower event rates among sustained users (70–72). However, these findings should not be interpreted as evidence that NSAIDs are cardioprotective.

Several forms of bias may influence these observations, including confounding by indication, survivor bias, treatment persistence bias, and selection of patients who tolerate long-term NSAID therapy. Therefore, NSAIDs should retain a double-edged interpretation in AS/axSpA: inflammation control may theoretically modify vascular risk, but this does not eliminate the known cardiovascular, renal, and gastrointestinal risks of NSAIDs, particularly in patients with established cardiovascular disease or high baseline risk (70–73). Drug choice, dose, duration, and monitoring should be individualized.

### 8.4 Evidence for JAK inhibitors in AS/axSpA remains limited, and caution is warranted

The use of JAK inhibitors in AS/axSpA is relatively recent. Current trial and comparative safety data in axial spondyloarthritis have not shown a definitive excess signal for major adverse cardiovascular events, but follow-up remains limited, event numbers are small, and confidence in long-term cardiovascular safety is still evolving (61, 68, 74, 75). Concerns regarding major adverse cardiovascular events and venous thromboembolism with JAK inhibitors are derived largely from rheumatoid arthritis and broader immune-mediated inflammatory disease datasets rather than from AS/axSpA-specific long-term outcome studies (76). Therefore, extrapolation to AS/axSpA should be cautious. At present, JAK inhibitors are best regarded as agents for which neither clear cardiovascular benefit nor fully established long-term cardiovascular safety has yet been demonstrated in AS/axSpA. In older patients, smokers, and those with established coronary disease, prior stroke, prior venous thromboembolism, or multiple cardiovascular risk factors, treatment selection should be individualized and accompanied by closer lipid and cardiovascular monitoring (61, 68, 75).

### 8.5 Other therapies and concomitant medications

Long-term glucocorticoid use is relatively uncommon in AS/axSpA, but whenever glucocorticoids or other therapies with metabolic liability are used, cardiovascular and metabolic adverse effects should be minimized as much as possible (77). Statins, although not antirheumatic therapies, remain important in patients who meet conventional indications for lipid lowering. In selected patients with persistent inflammation and accumulating vascular risk, clinicians may reasonably adopt a lower threshold for preventive cardiovascular discussion rather than assuming that young age or apparently modest LDL levels imply low risk (4, 56, 77).

More broadly, general biologic safety frameworks and pooled safety analyses may help guide treatment selection in patients with substantial comorbidity burden, even when the available data are not cardiovascular-endpoint specific (66, 77, 78). This is especially relevant in AS/axSpA, where drug choice often needs to balance musculoskeletal efficacy, extra-articular disease control, metabolic risk, and overall cardiovascular context. The available evidence regarding the potential cardiovascular effects of pharmacologic therapies in AS/axSpA is summarized in Table 5.

**Table 5:** Potential cardiovascular effects of pharmacologic therapies in ankylosing spondylitis and axial spondyloarthritis.

Therapy	Key study/exposure definition	Representative quantitative evidence	Evidence strength	Current interpretation and main caution
TNF inhibitors	Kwon and Park (26): retrospective axSpA cohort; exposure defined as TNFi use at index date and throughout follow-up. Other registry studies/meta-analyses also support possible benefit (22–25, 64).	450 axSpA patients; 233 TNFi-exposed; 20 CV events during 2,868 person-years. HR 0.30 (95% CI 0.10–0.85) after traditional risk-factor adjustment, attenuated after ESR/CRP adjustment (HR 0.37, 95% CI 0.12–1.12) and non-significant after IPTW (HR 0.60, 95% CI 0.23–1.54).	Moderate	Best-supported drug class for possible CV benefit, probably through inflammation control rather than a fixed cardioprotective dose. Interpret observational findings with confounding by indication in mind; individualize in pre-existing heart failure or cardiac vulnerability.
IL-17 inhibitors	Pooled safety and comparative studies in immune-mediated diseases and AS/axSpA; AS-specific hard-outcome follow-up remains limited (60, 62, 68, 69).	Large case-time-control data found no significant early MACE association after IL-17(R)A inhibitor initiation; long-term AS/axSpA event estimates remain insufficient.	Low to moderate	Acceptable short- to mid-term CV safety signal, but no proven long-term cardioprotection.
NSAIDs	Recent real-world AS analyses assessed long-term exposure and dose intensity (70–73).	High-dose versus low-dose NSAID exposure was associated with higher incident ischemic heart disease (aHR 1.08, 95% CI 1.05–1.11), stroke (aHR 1.09, 95% CI 1.04–1.15), and congestive HF (aHR 1.12, 95% CI 1.08–1.16) (70).	Moderate	Observational associations are complex and do not eliminate known NSAID CV, renal, and gastrointestinal risks. Use the lowest effective dose compatible with disease control, especially in high baseline CV risk.
JAK inhibitors	Upadacitinib and tofacitinib trial/safety datasets in AS/axSpA are relatively short-term and event-poor (6, 68, 74, 75).	Upadacitinib integrated programme: AS subgroup n = 182; no MACE and one VTE event. Tofacitinib integrated AS analysis reported no new safety signal over trial follow-up, but event numbers were small.	Low	No definitive excess MACE signal has been established in AS/axSpA, but RA-derived MACE/VTE warnings should be extrapolated cautiously. Monitor lipids and avoid complacency in older, smoking, or high-risk patients.
Glucocorticoids	Not a standard long-term AS/axSpA therapy; relevant mainly for selected indications or comorbid conditions (77).	No established AS/axSpA-specific CV benefit; chronic exposure is generally associated with metabolic and vascular liability in inflammatory disease care.	Low for AS/axSpA-specific evidence	Should not be considered cardioprotective; minimize dose and duration when used.
Statins and cardiometabolic therapies	Used according to general prevention indications; AS/axSpA-specific CV endpoint trials are lacking.	No AS/axSpA-specific randomized cardiovascular prevention trial establishes unique thresholds. General lipid/BP/glucose prevention evidence should be applied and individualized.	High for general prevention; low for AS/axSpA-specific evidence	Important adjunctive therapy when conventional risk, plaque burden, diabetes, or established CVD warrants treatment.

aHR, adjusted hazard ratio; AS, ankylosing spondylitis; axSpA, axial spondyloarthritis; BP, blood pressure; CI, confidence interval; CRP, C-reactive protein; CV, cardiovascular; CVD, cardiovascular disease; ESR, erythrocyte sedimentation rate; HF, heart failure; HR, hazard ratio; IPTW, inverse probability of treatment weighting; JAK, Janus kinase; MACE, major adverse cardiovascular events; RA, rheumatoid arthritis; TNF, tumor necrosis factor; TNFi, TNF inhibitor; VTE, venous thromboembolism. Evidence strength was graded pragmatically for this narrative review: high = meta-analysis or large population-based cohort with adjusted estimates; moderate = multicenter cohort, registry study, or consistent observational evidence; low = single-center, surrogate-only, short-term, or indirect evidence.

## 9 Clinical management: from symptom control to systemic risk reduction

### 9.1 Cardiovascular risk assessment should be a routine component of AS/axSpA care

Management of AS/axSpA can no longer be limited to musculoskeletal symptoms. Patients should undergo systematic assessment of blood pressure, lipids, glucose metabolism, body weight, smoking status, family history, and prior cardiovascular disease. Even younger patients may warrant early integration into a long-term prevention framework if they have persistent inflammatory activity, long disease duration, extra-articular manifestations, or clustering of comorbidities (3, 4, 21, 26).

Current guideline documents support a broader management approach, but they do not provide an AS/axSpA-specific cardiovascular risk calculator. The 2022 ASAS-EULAR recommendations emphasize individualized, multidisciplinary management and systematic consideration of comorbidities in axSpA, while EULAR cardiovascular risk management recommendations for inflammatory joint disorders recognize increased cardiovascular risk in AS and psoriatic arthritis and support regular screening and treatment of modifiable risk factors (79, 80). Conventional calculators such as SCORE2 or QRISK3 may be used as starting points for primary prevention discussions, but they were not calibrated specifically for AS/axSpA and do not directly capture cumulative inflammatory burden, persistent CRP elevation, extra-articular disease, or biologic exposure (81, 82). Therefore, calculator output should be interpreted conservatively in patients with sustained inflammation, long disease duration, uveitis, psoriasis, inflammatory bowel disease, or multimorbidity, rather than used as a stand-alone decision tool.

### 9.2 Lifestyle intervention remains the foundation and the most cost-effective strategy

Lifestyle management has dual benefits in AS/axSpA: it may improve pain, mobility, spinal function, and physical performance while also helping to control blood pressure, lipid status, glucose metabolism, body weight, and overall cardiovascular fitness. Low physical activity should be viewed not merely as a consequence of axial pain and stiffness, but also as an independent and modifiable contributor to cardiovascular risk. Structured aerobic exercise, combined with flexibility and strengthening programs, may improve functional capacity, support weight control, and potentially benefit endothelial function and vascular health. Core measures therefore include smoking cessation, weight control, regular aerobic and strengthening exercise, balanced diet, and psychological support (4, 45, 56, 79). Mental health also deserves attention, because depression and anxiety may indirectly amplify cardiovascular risk through unhealthy behaviors, reduced adherence, and autonomic stress.

### 9.3 Inflammation control is a form of cardiovascular protection

Disease activity should be brought as early as possible to low disease activity or remission. Persistently high inflammatory burden contributes directly to endothelial dysfunction, vascular remodeling, and future cardiovascular events (9, 23–26). This is why treatment adherence and sustained inflammation control should be emphasized in any review of AS/axSpA management: they influence not only pain and function, but also the long-term probability of myocardial infarction, stroke, heart failure, and other cardiovascular outcomes. Although AS/axSpA-specific adherence studies linked directly to cardiovascular endpoints remain limited, broader chronic disease data support the clinical importance of adherence as a determinant of long-term outcomes (83).

### 9.4 Cardiac monitoring and multidisciplinary care

Patients with cardiac murmurs, arrhythmia, dyspnea, long-standing disease, or other features suggestive of cardiac involvement may warrant electrocardiography and echocardiographic evaluation. Once aortic regurgitation or myocardial dysfunction is identified, follow-up intervals should be individualized according to severity, symptoms, and overall cardiovascular context (15, 17, 34, 50, 52). These patients are especially suitable for shared management involving rheumatology, cardiology, and where appropriate endocrinology or primary care, because cardiovascular risk in AS/axSpA is never a single-specialty issue. A practical approach to cardiovascular risk assessment and management in patients with AS/axSpA is proposed in Table 6.

**Table 6:** Practical cardiovascular assessment and management in patients with AS/axSpA.

Domain	What to assess	Operational/quantitative detail	Evidence strength	Suggested action
Traditional CV risk factors	Blood pressure, lipids, glucose/HbA1c, BMI or waist, smoking, family history, prior CVD.	Use a general-population calculator such as SCORE2 or QRISK3 as an initial framework; interpret cautiously when inflammatory burden is persistent.	High for conventional risk factors; moderate for AS/axSpA-specific modification	Assess at baseline and periodically. Treat hypertension, dyslipidemia, diabetes, obesity, and smoking according to standard prevention guidance.
Inflammatory burden	ASDAS-CRP/BASDAI, CRP, ESR, duration of active disease, response to therapy.	Sustained CRP elevation rather than a single measurement may identify higher vascular-risk exposure; 40% sustained CRP elevation was reported in one axSpA endothelial dysfunction study (9).	Moderate	Aim for early and sustained low disease activity; consider closer CV monitoring when inflammation remains persistently active.
Extra-articular disease	Uveitis, psoriasis, inflammatory bowel disease, and multimorbidity.	Uveitis was associated with ACS (aHR 1.675) (20) and stroke (competing-risk aHR 1.846) (38) in AS cohorts.	Moderate	Use extra-articular inflammatory manifestations as clinical warning signals for more careful CV assessment.
Lifestyle risk	Smoking, physical inactivity, obesity, diet quality, psychosocial stress.	Low physical activity should be considered both a consequence of axial symptoms and an independent CV risk factor; aerobic exercise also supports weight and endothelial health.	Moderate	Prescribe smoking cessation, structured aerobic activity plus mobility/strengthening work, weight management, and diet counselling.
Subclinical vascular assessment	cIMT/plaque, FMD or microvascular endothelial function, PWV/arterial stiffness when available.	cIMT was higher in r-axSpA than controls (0.8 +/− 0.1 versus 0.7 +/− 0.1 mm) (10); plaque/vascular markers are more useful in selected high-risk patients than universal screening.	Moderate for cIMT/plaque; low for routine biomarker use	Consider in patients with persistent inflammation, multiple risk factors, or uncertainty after standard risk calculation.
Rhythm assessment	Palpitations, syncope, dizziness, unexplained dyspnea, conduction abnormalities.	Registry data suggest increased AF and conduction disorder risk in AS; AF HR around 1.3 and AV block HR around 2.3 in AS versus the general population.	Moderate	Perform ECG when symptoms or examination suggest arrhythmia; consider ambulatory monitoring when symptoms are intermittent.
Structural cardiac evaluation	Murmur, dyspnea, reduced exercise tolerance, long disease duration, suspected aortic regurgitation or ventricular dysfunction.	Diastolic dysfunction is more frequent in axSpA (OR 3.43), whereas LVEF changes are small; assess E/A, e-prime, E/e-prime, LAVI and valve/aortic root structure rather than relying on LVEF alone (17).	Moderate	Use echocardiography selectively for symptoms, murmurs, long-standing disease, or previous abnormalities; individualize follow-up interval.
Multidisciplinary care	Established CVD, multiple risk factors, diabetes, CKD, heart failure, arrhythmia, or abnormal echocardiography.	No AS/axSpA-specific CV risk calculator is validated; multidisciplinary review is most justified when conventional risk and inflammatory risk accumulate.	Expert consensus plus moderate observational support	Coordinate rheumatology, cardiology, primary care, and endocrinology when needed; document shared prevention targets.

ACS, acute coronary syndrome; AF, atrial fibrillation; AS, ankylosing spondylitis; ASDAS, Ankylosing Spondylitis Disease Activity Score; axSpA, axial spondyloarthritis; BASDAI, Bath Ankylosing Spondylitis Disease Activity Index; BMI, body mass index; CI, confidence interval; cIMT, carotid intima-media thickness; CKD, chronic kidney disease; CRP, C-reactive protein; CV, cardiovascular; CVD, cardiovascular disease; ECG, electrocardiography; ESR, erythrocyte sedimentation rate; FMD, flow-mediated dilation; HR, hazard ratio; LAVI, left atrial volume index; LVEF, left ventricular ejection fraction; PWV, pulse wave velocity; r-axSpA, radiographic axial spondyloarthritis. Evidence strength was graded pragmatically for this narrative review: high = meta-analysis or large population-based cohort with adjusted estimates; moderate = multicenter cohort, registry study, or consistent observational evidence; low = single-center, surrogate-only, short-term, or indirect evidence.

## 10 Future directions

Future research on cardiovascular involvement in AS/axSpA should move beyond broad descriptions of excess risk toward testable and clinically actionable questions. The first priority is to develop and validate cardiovascular risk prediction strategies calibrated specifically for AS/axSpA. Conventional risk calculators should be evaluated against observed event rates, and candidate disease-related modifiers should include cumulative CRP burden, ASDAS or BASDAI trajectories, disease duration, extra-articular manifestations, smoking, metabolic comorbidities, carotid plaque, cIMT, arterial stiffness, and treatment exposure (6, 21).

Second, mechanistic studies should determine whether sustained inflammatory activity produces reproducible vascular phenotypes that predict later clinical events. The IL-6, CDCP1, and PON3 biomarker pattern discussed above should be validated in prospective cohorts and tested against endothelial dysfunction, cIMT progression, plaque formation, pulse wave velocity, and major cardiovascular events (9). The roles of IL-17-related pathways, HLA-B27-associated aortic root remodeling, and microbiome-related mechanisms should be treated as specific hypotheses requiring longitudinal and translational confirmation rather than as established explanations (52, 63).

Third, comparative treatment studies should be designed around explicit cardiovascular hypotheses. For example, among patients with high inflammatory burden, active-comparator new-user studies or pragmatic trials could test whether TNF inhibitors differ from IL-17 inhibitors or JAK inhibitors in their effects on vascular surrogate markers and hard cardiovascular outcomes. Such studies should define exposure windows, cumulative treatment duration, dose where available, time-varying inflammation, and baseline cardiovascular risk to reduce confounding by indication and immortal-time bias (22, 62, 68).

Fourth, future clinical research should define feasible monitoring pathways rather than simply recommending general vigilance. Studies should compare strategies such as annual cardiovascular risk reassessment, inflammation-adjusted interpretation of SCORE2 or QRISK3, targeted carotid ultrasound or pulse wave velocity testing in high-risk patients, echocardiography for long-standing disease or murmurs, and multidisciplinary rheumatology-cardiology management. Implementation outcomes, including screening uptake, treatment intensification, patient adherence, cost, and event reduction, should be evaluated alongside biological endpoints.

Finally, digital health tools and emerging anti-inflammatory delivery platforms may support more personalized long-term monitoring, but their value in AS/axSpA cardiovascular prevention should be tested through predefined endpoints rather than assumed from general inflammatory arthritis experience (84, 85). The proposed research agenda is summarized in Table 7.

**Table 7:** Operational research priorities for cardiovascular involvement in AS/axSpA.

Priority area	Operational research question and hypothesis	Suggested design and endpoints
Risk prediction	Do conventional calculators underestimate risk in AS/axSpA? Hypothesis: inflammation-related modifiers improve calibration.	Prospective cohort and external validation; calibration, C-statistic, net reclassification; MACE, stroke, myocardial infarction.
Biomarkers	Can cumulative CRP or IL-6/CDCP1/PON3 identify early vascular injury? Hypothesis: longitudinal inflammatory burden predicts vascular dysfunction.	Repeated biomarker-imaging cohort; time-weighted CRP, FMD, cIMT, carotid plaque, PWV, MACE.
Mechanisms	Which pathways link IL-17, HLA-B27-related aortic root remodeling, microbiome changes, and endothelial dysfunction?	Translational cohort; cytokines, proteomics, microbiome data, vascular imaging, echocardiography, valve and aortic root measures.
Treatment effects	Do TNFi, IL-17i, JAKi, and NSAID exposure differ in cardiovascular effects after adjustment for inflammation control?	Active-comparator new-user or target-trial emulation; MACE, VTE, heart failure, AF, lipid changes, cIMT/PWV/FMD.
Implementation	Which cardiovascular screening pathway is feasible and improves routine AS/axSpA care?	Cluster-randomized or stepped-wedge study; screening uptake, preventive treatment, lifestyle change, referral, cost-effectiveness, event reduction.

## 11 Conclusion

The present review highlights that cardiovascular involvement in ankylosing spondylitis and axial spondyloarthritis should no longer be regarded as a secondary or incidental issue, but rather as a clinically meaningful component of the overall disease burden (4–6). Available evidence indicates that patients with AS/axSpA have increased risks of major cardiovascular events together with a broad spectrum of vascular and cardiac abnormalities, even in the era of biologic therapy (6, 17, 22, 25). Taken together, these findings support a broader interpretation of AS/axSpA as a systemic inflammatory disease with important cardiovascular consequences rather than a condition confined to the axial skeleton (4–6).

A key message from the current literature is that cardiovascular injury in AS/axSpA often begins during a prolonged subclinical phase. Vascular dysfunction and early structural abnormalities may accumulate for years before myocardial infarction, stroke, or overt heart failure becomes clinically apparent (7, 10–13). Clinically, this suggests that reliance on hard cardiovascular endpoints alone may underestimate the true burden of disease and that preventive efforts should begin earlier, before irreversible organ damage has developed (7, 11, 13).

Another important point is that cardiovascular risk in AS/axSpA is heterogeneous rather than uniform (2, 3, 18, 19, 21). Patients with sustained inflammatory activity, persistently elevated CRP, long disease duration, extra-articular manifestations, multiple comorbidities, suboptimal disease control, or difficult-to-treat disease appear to carry a substantially greater burden (2, 3, 9, 20, 38). This heterogeneity argues against a one-size-fits-all approach and suggests that inflammatory burden should be considered alongside conventional cardiovascular risk assessment (3, 18, 19, 21).

The relationship between traditional cardiovascular risk factors and inflammation is central to understanding this excess risk. Hypertension, dyslipidemia, abnormal glucose metabolism, obesity, smoking, and metabolic syndrome remain highly relevant, but their effects are modified by chronic inflammation (4, 45, 50, 55, 56). In particular, the inflammatory lipid paradox and the association of persistent inflammation with endothelial dysfunction, arterial stiffness, oxidative stress, and vascular remodeling indicate that conventional risk factors provide the substrate, whereas inflammation accelerates progression toward clinically relevant cardiovascular disease (7, 9, 12, 19, 55).

From a therapeutic perspective, current evidence most strongly supports TNF inhibitors as the drug class with the clearest potential for cardiovascular benefit, likely through more effective suppression of systemic inflammation rather than a direct cardioprotective effect (22–26). By contrast, the long-term cardiovascular implications of IL-17 inhibitors, NSAIDs, and JAK inhibitors remain less certain and should be interpreted cautiously; for JAK inhibitors, concerns regarding MACE and venous thromboembolism are still largely extrapolated from rheumatoid arthritis and broader immune-mediated disease datasets rather than established AS/axSpA-specific long-term evidence (60, 62, 68–76). These observations reinforce a practical principle: in AS/axSpA, inflammation control should be regarded as part of cardiovascular prevention, but it does not replace the need for active management of conventional risk factors and individualized monitoring in selected patients (4, 21, 25, 26, 50).

This review also has limitations inherent to its narrative design. Much of the current literature remains observational, and causal inference is constrained by confounding, treatment selection bias, heterogeneity in disease definitions, and variable outcome ascertainment (6, 21, 22, 62, 68). In addition, many studies focus on surrogate vascular markers rather than hard cardiovascular endpoints, and randomized comparisons of targeted therapies for cardiovascular outcomes are still lacking (10–12, 17, 34). These gaps likely explain persistent inconsistencies across studies, particularly regarding mortality, valvular disease prevalence, and comparative drug effects (6, 8, 17, 52). Overall, however, the available evidence supports a conceptual shift in management: cardiovascular disease in AS/axSpA should be viewed as part of the systemic consequences of persistent inflammation interacting with conventional risk factors over time (4–6). Until disease-specific risk models and stronger long-term comparative data become available, a pragmatic strategy combining early inflammation control, aggressive modification of modifiable risk factors, individualized cardiovascular monitoring, and careful attention to broader multimorbidity remains the most reasonable approach (6, 21, 62, 68).
