# Supplementary material for: Cardiovascular involvement in ankylosing spondylitis and axial spondyloarthritis: epidemiology, mechanisms, and clinical management
Source: Front Med (Lausanne). 2026 May 18;13:1841913. doi: 10.3389/fmed.2026.1841913 (PMC13222810; doi:10.3389/fmed.2026.1841913)
Supplement: Supplementary file 1 [file Table_1.docx]

**Supplementary Table 1. Representative database search strategy**

The search strategy was adapted for the syntax of each database. Searches covered records from database inception to January 31, 2026 and were restricted to English-language human studies. The primary searches used broad disease-related and cardiovascular terms. Mechanistic and treatment-related terms were used in supplementary topic-specific searches rather than as mandatory filters for all records, so that epidemiologic, imaging, and clinical outcome studies would not be excluded simply because they did not mention mechanisms or therapies in the title or abstract.

| **Database** | **Representative search strategy** | **Limits and notes** |
| --- | --- | --- |
| PubMed | **Primary clinical/vascular search:**  (("Spondylitis, Ankylosing"[MeSH Terms] OR "ankylosing spondylitis"[tiab] OR "axial spondyloarthritis"[tiab] OR "axial SpA"[tiab] OR "radiographic axial spondyloarthritis"[tiab] OR "non-radiographic axial spondyloarthritis"[tiab] OR "nonradiographic axial spondyloarthritis"[tiab] OR axSpA[tiab]) AND ("Cardiovascular Diseases"[MeSH Terms] OR "cardiovascular disease"[tiab] OR "cardiovascular risk"[tiab] OR "major adverse cardiovascular event"[tiab] OR "major adverse cardiovascular events"[tiab] OR MACE[tiab] OR "myocardial infarction"[tiab] OR stroke[tiab] OR "atrial fibrillation"[tiab] OR "heart failure"[tiab] OR atherosclerosis[tiab] OR "endothelial dysfunction"[tiab] OR "arterial stiffness"[tiab] OR "pulse wave velocity"[tiab] OR "carotid intima-media thickness"[tiab] OR "carotid intima media thickness"[tiab] OR cIMT[tiab] OR "carotid plaque"[tiab] OR "aortic regurgitation"[tiab] OR "valvular disease"[tiab] OR "valvular heart disease"[tiab]))  **Supplementary topic-specific searches:**  The same disease block was combined with selected mechanism or therapy terms, including inflammation, "C-reactive protein", CRP, "tumor necrosis factor", TNF, "interleukin-17", IL-17, "interleukin-6", IL-6, "TNF inhibitor", "IL-17 inhibitor", "Janus kinase inhibitor", biologic*, "non-steroidal anti-inflammatory drug", and NSAID*. These supplementary terms were added where appropriate; they were not applied as a mandatory third AND block to the primary clinical/vascular search. | English; humans; inception to January 31, 2026.  No automatic study-design filter was applied.  Standalone AS[tiab] was avoided to reduce false retrieval; disease abbreviations were searched only when sufficiently specific, such as axSpA. |
| Embase | **Primary clinical/vascular search:**  ('ankylosing spondylitis'/exp OR 'ankylosing spondylitis':ti,ab OR 'axial spondyloarthritis':ti,ab OR 'axial spa':ti,ab OR 'radiographic axial spondyloarthritis':ti,ab OR 'non-radiographic axial spondyloarthritis':ti,ab OR 'nonradiographic axial spondyloarthritis':ti,ab OR axspa:ti,ab) AND ('cardiovascular disease'/exp OR 'cardiovascular risk':ti,ab OR 'major adverse cardiovascular event':ti,ab OR 'major adverse cardiovascular events':ti,ab OR mace:ti,ab OR 'myocardial infarction'/exp OR 'myocardial infarction':ti,ab OR stroke/exp OR stroke:ti,ab OR 'atrial fibrillation'/exp OR 'atrial fibrillation':ti,ab OR 'heart failure'/exp OR 'heart failure':ti,ab OR atherosclerosis/exp OR atherosclerosis:ti,ab OR 'endothelial dysfunction':ti,ab OR 'arterial stiffness':ti,ab OR 'pulse wave velocity':ti,ab OR 'carotid intima media thickness':ti,ab OR cimt:ti,ab OR 'carotid plaque':ti,ab OR 'aortic regurgitation':ti,ab OR 'valvular disease':ti,ab OR 'valvular heart disease':ti,ab)  **Supplementary topic-specific searches:**  The same disease block was combined with selected Emtree/free-text mechanism or therapy terms, including 'inflammation'/exp, 'c reactive protein'/exp, 'tumor necrosis factor'/exp, 'interleukin 17'/exp, 'interleukin 6'/exp, 'janus kinase inhibitor'/exp, 'biological therapy'/exp, 'tumor necrosis factor inhibitor'/exp, 'interleukin 17 inhibitor':ti,ab, 'nonsteroid antiinflammatory agent'/exp, biologic*:ti,ab, and nsaid*:ti,ab, with cardiovascular outcome or vascular terms added where relevant. These terms were not used as a universal eligibility filter. | English; humans; inception to January 31, 2026.  Emtree terms and free-text terms were combined.  Primary searches were not restricted by study design; supplementary mechanism/treatment searches were used only for topic-specific sections. |
| Web of Science | **Primary clinical/vascular search:**  TS=("ankylosing spondylitis" OR "axial spondyloarthritis" OR "axial SpA" OR "radiographic axial spondyloarthritis" OR "non-radiographic axial spondyloarthritis" OR "nonradiographic axial spondyloarthritis" OR axSpA) AND TS=("cardiovascular disease" OR "cardiovascular risk" OR "major adverse cardiovascular event*" OR MACE OR "myocardial infarction" OR stroke OR "atrial fibrillation" OR "heart failure" OR atherosclerosis OR "endothelial dysfunction" OR "arterial stiffness" OR "pulse wave velocity" OR "carotid intima-media thickness" OR "carotid intima media thickness" OR cIMT OR "carotid plaque" OR "aortic regurgitation" OR "valvular disease" OR "valvular heart disease")  **Supplementary topic-specific searches:**  TS=(inflammation OR "C-reactive protein" OR CRP OR TNF OR "tumor necrosis factor" OR "interleukin-17" OR IL-17 OR "interleukin-6" OR IL-6 OR "TNF inhibitor*" OR "IL-17 inhibitor*" OR "Janus kinase inhibitor*" OR biologic* OR NSAID* OR "non-steroidal anti-inflammatory drug*") was combined with the disease block and, where relevant, selected cardiovascular outcome or vascular terms. These topic-specific searches supplemented rather than replaced the broad primary search. | English; inception to January 31, 2026.  Used to identify additional cohort, imaging, citation-linked, mechanistic, and treatment-safety publications.  Mechanistic/treatment terms were not required for inclusion in the broad clinical/vascular evidence base. |

Abbreviations: AS, ankylosing spondylitis; axSpA, axial spondyloarthritis; cIMT, carotid intima-media thickness; CRP, C-reactive protein; IL, interleukin; JAK, Janus kinase; MACE, major adverse cardiovascular events; NSAID, non-steroidal anti-inflammatory drug; TNF, tumor necrosis factor.
